# A comparison of RNA extraction and sequencing protocols for detection of small RNAs in plasma

**DOI:** 10.1186/s12864-019-5826-7

**Published:** 2019-06-03

**Authors:** Ryan K.Y. Wong, Meabh MacMahon, Jayne V. Woodside, David A. Simpson

**Affiliations:** 1 Centre for Experimental Medicine, School of Medicine, Dentistry and Biomedical Sciences, Queen’s University Belfast, 97 Lisburn Road, Belfast, BT9 7BL UK; 2 Nutrition Group, Institute for Global Food Security (Centre for Public Health), School of Medicine, Dentistry and Biomedical Sciences, Queen’s University Belfast, Institute of Clinical Science A (First Floor), Grosvenor Road, Belfast, BT12 6BJ UK

**Keywords:** microRNA, miRNA, Small RNA-Seq, Circulating biomarker, Next generation sequencing, NGS, Plasma, Library preparation

## Abstract

**Background:**

Circulating microRNAs (miRNAs) are attractive non-invasive biomarkers for a variety of conditions due to their stability and altered pathophysiological expression levels. Reliable detection of global expression profiles is required to maximise miRNA biomarker discovery. Although developments in small RNA-Seq technology have improved detection of plasma-based miRNAs, the low RNA content and sequencing bias introduced during library preparation remain challenging. In this study we compare commercially available RNA extraction methods using MagnaZol (Bioo Scientific) or miRNeasy (QIAGEN) and three library preparation methods - CleanTag (TriLink), NEXTflex (Bioo Scientific) and QIAseq (QIAGEN) - which aim to address one or both of these issues.

**Results:**

Different RNA extractions and library preparation protocols result in differential detection of miRNAs. A greater proportion of reads mapped to miRNAs in libraries prepared with MagnaZol RNA than with miRNeasy RNA. Libraries prepared using QIAseq demonstrated the greatest miRNA diversity with many more very low abundance miRNAs detected (~ 2–3 fold more with < 10 reads), whilst CleanTag detected the fewest individual miRNAs and considerably over-represented miR-486-5p. Libraries prepared with QIAseq had the strongest correlation with RT-qPCR quantification. Analysis of unique molecular indices (UMIs) incorporated in the QIAseq protocol indicate that little PCR bias is introduced during small RNA library preparation.

**Conclusions:**

Small RNAs were consistently detected using all RNA extraction and library preparation protocols tested, but with some miRNAs at significantly different levels. Choice of the most suitable protocol should be informed by the relative importance of minimising the total sequencing required, detection of rare miRNAs or absolute quantification.

**Electronic supplementary material:**

The online version of this article (10.1186/s12864-019-5826-7) contains supplementary material, which is available to authorized users.

## Background

MicroRNAs (miRNAs) are attractive biomarkers because they can reflect tissue state and are stable in biofluids [1]. The ready availability of blood samples has driven the development of plasma miRNAs as clinical biomarkers for detection of cancer [2, 3] and a range of other conditions [4–6]. It has been suggested that detection of miRNAs indicative of specific organs could form the basis of a universal test to determine the site of pathology [7].

Global miRNA profiling is often used as a discovery tool to detect specific new miRNA biomarkers which are subsequently detected using RT-qPCR, which remains the gold standard for measuring individual or selected groups of miRNAs. Next generation sequencing (NGS) has become the principal approach for global profiling of miRNAs because it is potentially more sensitive than microarrays and has the advantage that target sequences do not need to be known in advance. However, wider adoption of NGS to detect miRNAs and other small RNAs (small RNA-Seq) is hampered by biases which mean that the expression values measured for miRNAs may not accurately reflect their absolute levels. Sequencing bias is introduced during library preparation, primarily during adapter ligation. It has been shown that ligation bias is determined by preferential secondary structures formed between miRNAs and adapters and that it can be reduced by use of adapters with degenerate bases [8–11].

The low concentration of miRNAs in plasma also presents a challenge for library preparation [12–15], particularly from small volumes. Low RNA input can result in a high proportion of adapter dimer and non-miRNA reads with a concomitant reduction in the number of reads mapping to miRNAs, which necessitates greater raw sequencing depth. The many non-target reads often detected from exogenous RNAs likely reflect the greater proportion of contaminating RNA molecules in low input samples [16–18]. To investigate the possibility of diet as a source of exogenous miRNAs we included plasma samples from the same individuals before and after a change in diet to include considerably more plant material.

The development of simple, robust protocols that address bias and low RNA input would both facilitate miRNA biomarker discovery and increase the feasibility of using small RNA-Seq itself as a tool to generate miRNA profiles for use as biomarkers. Indeed, as sequencing costs decrease, facilitating higher throughput, RNA-Seq may become the primary technique for measuring circulating miRNAs.

Protocols for the preparation of small RNA-Seq libraries are continually improving [19, 20] and various aspects of the expanding number of alternatives have been reviewed [11, 21–24]. Issues particularly pertinent for blood-based biomarker discovery are the ability to work with low miRNA concentrations and reduction of bias. Here we assess the efficacy of three recently commercially available small RNA library preparation methods specifically designed to address one or both of these issues. CleanTag™ Small RNA Library Prep Kit (TriLink) uses modified adapters to reduce adapter dimer formation from low inputs of RNA [20]. NEXTflex® Small RNA Sequencing Kit v3 (Bioo Scientific) uses randomised adapters to reduce sequencing bias and adapter dimer reduction technology to allow low inputs of RNA [19]. QIAseq miRNA Library Kit (QIAGEN) claims to employ optimised reaction chemistry to reduce bias, minimise adapter dimer formation and contaminating non-miRNAs, facilitating low inputs of RNA. QIAseq is the only kit to incorporate unique molecular indices (UMIs) into each cDNA to enable correction for PCR bias.

RNA extraction methods have been reported to affect the profile of miRNAs detected [13, 15, 25, 26]. The MagnaZol™ cfRNA Isolation Reagent (Bioo Scientific) and miRNeasy Serum/Plasma kit (QIAGEN) are RNA extraction kits for the extraction of small RNAs from biofluids, specifically designed to work with low input volumes of plasma. We assess how RNA extracted with these kits performs with both library preparation kits supplied by the respective manufacturers. A workflow identifying the key differences between RNA extraction and library preparation methods is provided in Additional file 1.

Our results show that whilst all protocols provided reproducible results, which can be used for comparison of relative expression, the miRNA profile detected from plasma samples is greatly affected by the choice of library preparation kit and, to a lesser extent, the RNA extraction method.

## Results/discussion

### Study design

To compare the efficiency of RNA extraction methods, RNA was extracted from the plasma of three individuals at two time points. The time points were before and after a change in diet, increasing plant consumption, with an increase in fruit and vegetable intake from < 2 to 8 servings per day over 4 weeks in a controlled setting with all food provided and two meals per day consumed under supervision to maximise compliance [27]. RNA was extracted using either the MagnaZol™ cfRNA Isolation Reagent (M) from the maximum volume of 600 μL of plasma per 2 mL tube or the miRNeasy Serum/Plasma extraction (m) from the maximum volume of 200 μL of plasma per column. The miRNeasy extraction was carried out twice on each plasma sample and purified RNA was pooled to increase the volume of RNA available for library preparation.

Three library preparation kits, CleanTag (CT), NEXTflex (NF) and QIAseq (Q), were compared on all miRNeasy RNA samples because this extraction method has been shown previously to perform favourably against other commercially available kits [15]. For both mQ and mNF, 5 μL of RNA was used because this is the recommended input from serum/plasma for QIAseq, whilst NEXTflex has a variable input (up to 10.5 μL) but gives no recommendation for serum/plasma. For CleanTag, the maximum input of 2 μL of RNA was used. QIAseq and NEXTflex libraries were also prepared on 5 μL of MagnaZol RNA from the three individuals at two time points to compare library preparations on RNA extractions provided by both manufacturers. mCT, mQ and MQ libraries were purified using the recommended gel free magnetic bead cleanup (Agencourt AMPure XP beads for CT, QMN beads for Q). mNF and MNF libraries were purified using PAGE size selection as recommended for low input RNA. All libraries were prepared in the same laboratory by the same individual. A list of abbreviations outlining the RNA extraction and library preparation methods is provided in Table 1.

**Table 1:** – Abbreviations describing RNA extraction and library preparation methods

Abbreviation	RNA extraction method	Library preparation method
mNF	miRNeasy	NEXTflex
mQ	miRNeasy	QIAseq
mCT	miRNeasy	CleanTag
MNF	MagnaZol	NEXTflex
MQ	MagnaZol	QIAseq

### Effects of unique molecular indices (UMIs)

**Fig. 1:**
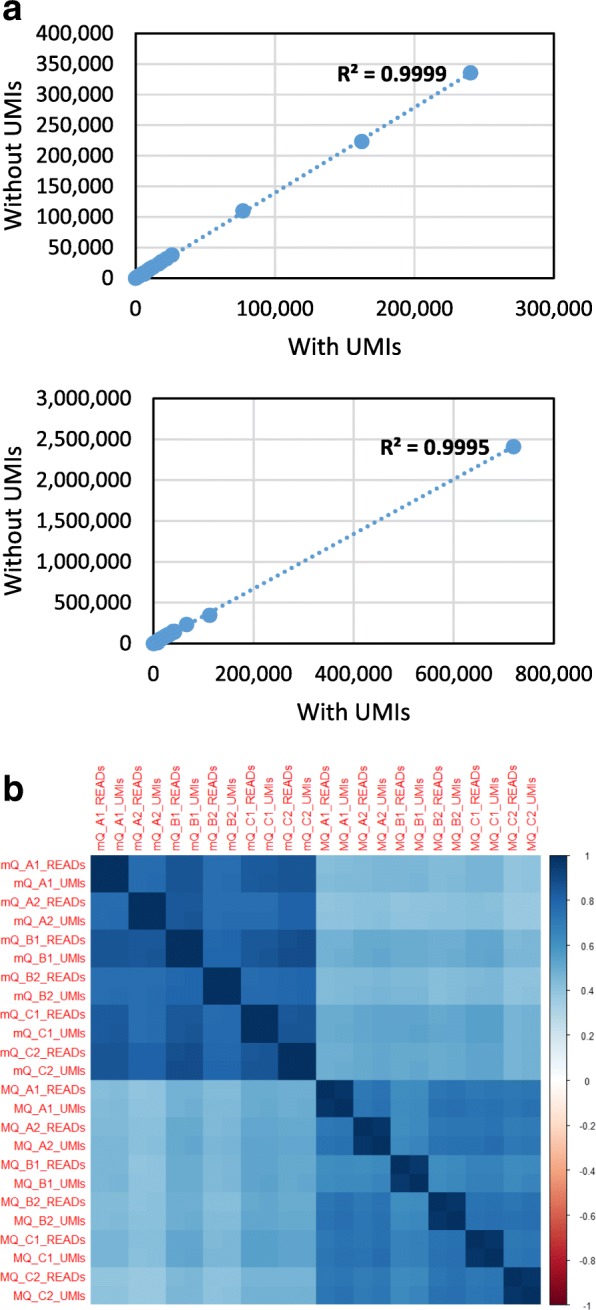
Effect of correction for PCR duplication using UMIs. **a** Scatter plot of the number of reads versus the number of unique UMIs mapping to each miRNA (values are the mean of 6 samples). **b** Correlation between all QIAseq miRNA profiles when analysed with (*_UMIs) or without (*_READS) UMI correction. The correlation between individual samples with and without UMI correction is extremely high (≥0.97).The correlations are higher between replicate samples prepared with the same RNA extractions

QIAseq is the only library preparation kit that uses UMIs to account for PCR bias. The reads mapping to miRNAs in all QIAseq libraries were calculated with and without UMI correction from raw reads downsampled to 5 million reads. Comparison of the mean number of reads with and without UMI correction showed a strong correlation for both mQ and MQ libraries (Fig. 1a). Visualisation of the correlation coefficients between all QIAseq libraries confirmed the similarity between the same libraries with and without UMI correction (≥0.97) and highlighted the higher correlation between libraries prepared using the same RNA extraction method (Fig. 1b). The similarity between the proportions of reads mapping to each miRNA with or without UMI correction suggests that there is little PCR bias introduced during library preparation. This agrees with previous studies which also showed that PCR bias is negligible [8, 10, 21] and likely reflects the very similar length of all miRNA-containing amplicons, which are being amplified using the same flanking primers. The QIAseq libraries were subsequently analysed without UMI correction to enable direct comparison with the other protocols.

Although the number of amplification cycles was the same for QIAseq libraries prepared on miRNeasy or Magnazol RNA (mQ and MQ), reads prior to UMI correction were 1.48 and 4.29 times higher respectively (see Fig. 1a). This suggests that there may have been more miRNAs in the miRNeasy-extracted input RNA. Assuming amplification was within the exponential phase this would result in a larger miRNA library, with the sample of reads sequenced representing a smaller proportion and therefore with fewer duplicated UMIs.

### Read mapping

**Fig. 2:**
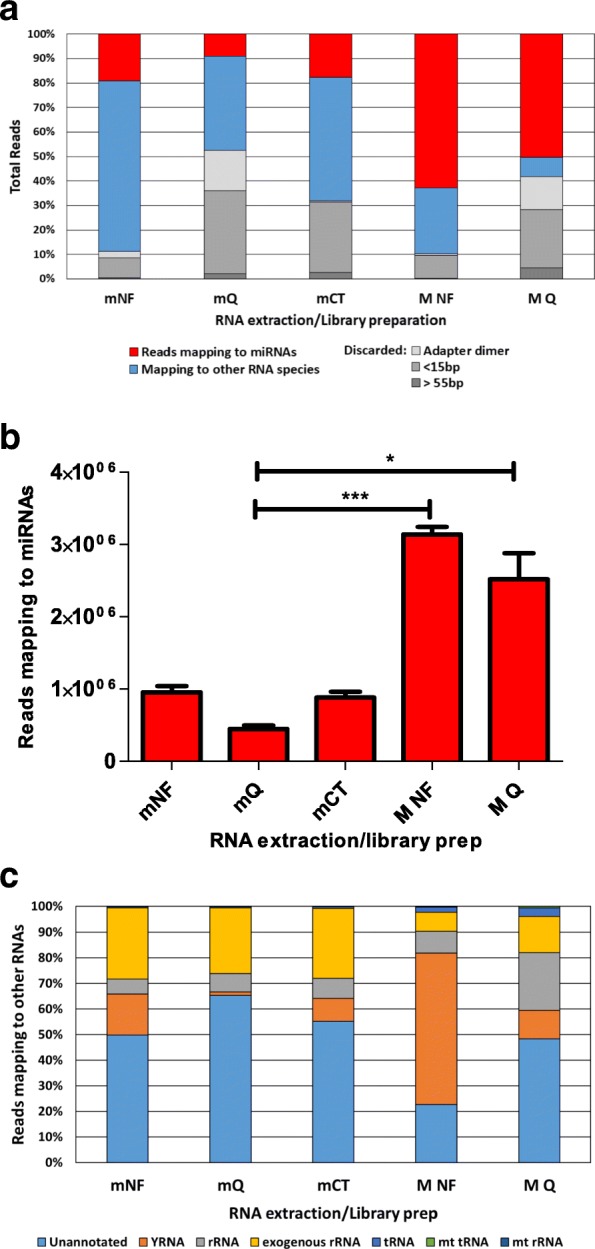
Read mapping. **a** Average proportion of reads mapping to miRNAs (red), other RNA species (blue) and discarded for being too long, short or adapter dimer (grey) in each RNA extraction/library preparation combination (*n* = 6). **b** Total reads were downsampled to 5 million and the number of reads mapping to miRNAs was determined for each RNA extraction and library preparation method (n = 6). Error bars show standard error of the mean, significance was determined using Friedman test and Dunn’s multiple comparison test. * *p* < 0.05 ***p < 0.001, where no *p*-value shown there was no significance. **c** Percentages of non-miRNA reads mapping to specific RNA types

The percentage of raw reads mapping to miRNAs, reads mapping to other RNAs and reads discarded (too long > 55 bp, too short < 15 bp and adapter dimer) were averaged for each RNA extraction and library preparation combination and compared between mNF, mQ, mCT, MNF and MQ (Fig. 2a). From the miRNeasy RNA, mNF had the highest percentage of reads mapping to miRNAs with an average of 18.9% and the lowest percentage of reads discarded at 11.4%. mCT had an average of 17.2% reads mapping to miRNAs and 31.4% of reads discarded. mQ had the lowest percentage of reads mapping to miRNAs with an average of 9.5% and the highest percentage of reads discarded at 50.6%. From the MagnaZol RNA, MNF had the highest percentage of reads mapping to miRNAs of all extraction/library combinations, with an average of 62.8% mapping and only 10.3% of reads discarded. MQ had an average of 50.3% reads mapping to miRNAs and 41.8% of reads discarded. MNF and MQ both detected a significantly higher proportion of miRNA reads compared to mQ (Fig. 2b). The higher percentage of reads mapping to miRNAs in libraries prepared from MagnaZol RNA suggests that miRNAs form a greater proportion of the RNAs present in these extractions that contribute to the libraries. CleanTag libraries had the lowest proportion of adapter dimers (0.4% of reads) and QIAseq libraries had the highest proportion (16% of mQ reads and 14% of MQ reads).

Reads not mapping to human miRNAs were aligned to other small RNA databases to determine the distribution of the remaining reads. While some remained unannotated, most mapped to ribosomal RNA (rRNAs) and Y RNAs (Fig. 2c). Many more reads from NEXTflex libraries mapped to the Y RNAs, which are specifically blocked in the QIAseq protocol. To assess the presence of exogenous plant RNAs, potentially of dietary origin, sequences not matching human miRNAs were aligned with all mature plant miRNAs from miRBase. Several sequences were identified (100% identity, > 17 nucleotides) and are listed in Additional file 2. Although many of these concur with previously reported plant miRNAs [28], their abundance did not demonstrate a consistent increase in individuals following a change to a high plant content diet (Additional file 3), suggesting that these are likely a result of contamination [17, 29]. No endogenous miRNAs demonstrated a consistent alteration in expression following the change to a high plant content diet.

### Library diversity

**Fig. 3:**
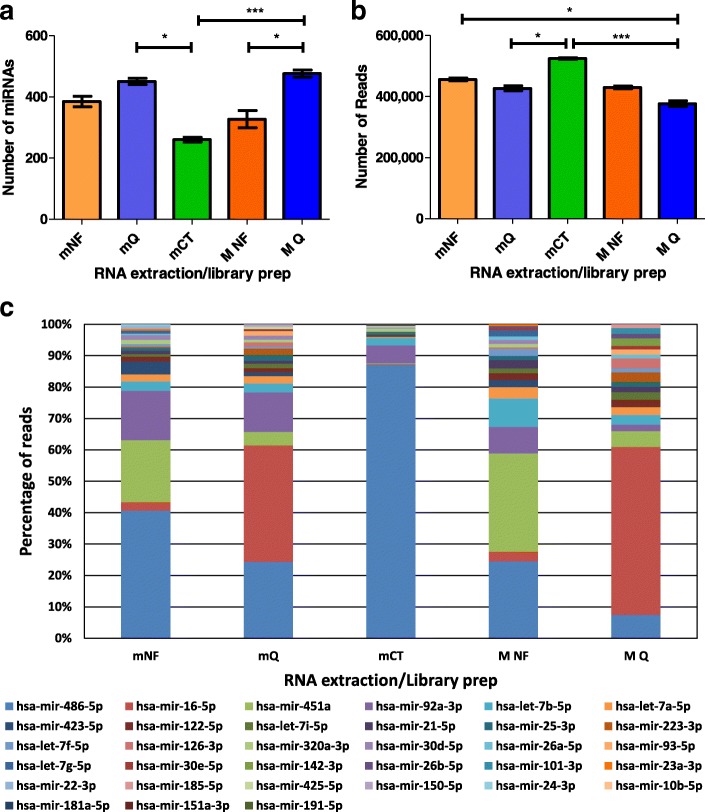
miRNA diversity. **a** The number of different miRNAs detected (≥ 2 reads) from an equal number of reads mapped to miRNAs (550,000). Error bars show standard error of the mean (n = 6), significance determined by applying Friedman test and Dunn’s Multiple Comparison test. **b** Number of reads mapping to the 10 most highly expressed miRNAs in each RNA extraction/library preparation combination. **c** Read distribution of the 20 most highly represented miRNAs from each RNA extraction/library preparation combination. * p < 0.05 ***p < 0.001 where no p-value shown there was no significance

Library diversity is indicative of bias, with over-representation of certain miRNAs resulting in lack of detection of other lowly expressed miRNAs. To compare the efficiency of each library prep kit at reducing bias, the number of individual miRNAs detected from each RNA extraction and library preparation combination was determined from an equal number of reads mapping to miRNAs. Reads mapping to miRNAs were extracted, downsampled to 550,000 reads and the number of miRNAs detected (with a minimum of 2 reads) in every sample for each RNA extraction and library preparation combination averaged (Fig. 3a). The highest number of individual miRNAs was detected in QIAseq libraries, with MQ detecting an average of 471 miRNAs and mQ detecting an average of 451 miRNAs. mNF detected an average of 385 miRNAs and MNF detected an average of 327 miRNAs. mCT detected the lowest number of miRNAs with an average of 260 miRNAs. Friedman and Dunn’s Multiple Comparison tests were applied and showed there were significant differences between the number of miRNAs detected between mQ and mCT, MQ and mCT and MQ and MNF (Fig. 3a).

To further compare library diversity the reads for each miRNA, detected from a total of 550,000 reads mapping to miRNAs, were averaged for each RNA extraction and library preparation combination. A similar pattern of relative library diversity to that suggested by the total number of miRNAs detected was revealed by comparing the number of reads assigned to the top 10 miRNAs in each protocol (Fig. 3b). The highest number of reads (i.e. least diversity) was recorded for mCT with 524,747 reads, while MQ had the lowest number of reads at 391961.

Over-representation of specific miRNAs in certain protocols is illustrated by comparing reads mapping to each of the 20 most highly expressed miRNAs. For example, miR-486-5p by CleanTag, miR-451a by NEXTflex and miR-16-5p by QIAseq (Fig. 3c).

### Differentially detected miRNAs between RNA extraction/library preparations

If a miRNA is differentially detected between different library preparations or RNA extractions it must be subject to under- or over-representation in at least one of the protocols. Similarity with quantification by an independent method, such as RT-qPCR, can suggest which measurement is most likely closest to the absolute value. To compare differences in miRNA detection between library preparations, a two-group paired comparison was performed between NF and Q libraries prepared on the same RNA extraction for both extractions (550,000 reads mapping to miRNAs). We considered further miRNAs significantly differentially detected (≥2 fold change and Bonferroni corrected *P*-values ≤0.05) between NF and Q in both RNA extractions; there were 18 miRNAs higher in NF than Q and 25 miRNAs higher in Q than NF. A similar comparison was performed for miRNAs differentially detected between RNA extractions; 10 miRNAs were higher by both library preparation methods in MagnaZol than miRNeasy and 2 miRNAs higher in miRNeasy than MagnaZol. Table 2 shows miRNAs differentially detected in the same direction between library preparations in both RNA extractions. A full list of differentially detected miRNAs between library preparations and RNA extractions is provided in Additional file 4.

**Table 2:** miRNAs differentially detected between NEXTflex and QIAseq library preparations. Significantly differentially detected miRNAs were identified by a two-group paired comparison (n = 6) between NEXTflex and QIAseq library preparations on both MagnaZol and miRNeasy RNA extractions, with Baggerley’s test and Bonferroni correction. miRNAs consistently differing between library preparations from MagnaZol and miRNeasy are listed, with positive fold change indicating higher detection in QIAseq and negative fold-change higher detection in NEXTflex

miRNA	MagnaZol Fold Change	MagnaZol p-value	miRNeasy Fold Change	miRNeasy *p*-value	Higher In
let-7d-3p	−9.6	8.88E-40	−3.8	1.64E-11	NF
let-7 g-5p	−3.1	2.82E-13	−3.0	8.66E-25	NF
let-7i-3p	18.1	4.67E-08	5.7	1.85E-03	Q
mir-103a-1-3p	2.1	6.69E-05	2.5	6.86E-04	Q
mir-103a-2-3p	2.1	8.77E-05	2.6	5.71E-04	Q
mir-10a-5p	−4.7	3.38E-11	−2.5	7.99E-08	NF
mir-10b-5p	−5.9	1.14E-23	−4.0	4.24E-07	NF
mir-1260b-5p	−20.8	1.46E-08	−8.5	2.85E-03	NF
mir-126-5p	9.6	5.66E-09	16.9	0.00	Q
mir-130a-3p	4.9	3.27E-08	4.5	4.74E-03	Q
mir-130b-5p	−11.1	1.42E-03	−6.3	6.83E-10	NF
mir-142-3p	71.7	0.00	40.2	3.53E-06	Q
mir-142-5p	11.5	8.96E-04	4.6	8.39E-06	Q
mir-148b-3p	2.7	5.36E-06	2.7	5.46E-08	Q
mir-151a-5p	−11.4	2.99E-07	−16.1	1.24E-07	NF
mir-152-3p	6.4	9.85E-06	4.8	1.79E-02	Q
mir-15a-5p	2.8	5.38E-03	3.1	3.59E-02	Q
mir-16-1-5p	15.1	0.00	13.2	0.00	Q
mir-16-2-3p	− 57.3	9.57E-28	−39.8	2.97E-52	NF
mir-16-2-5p	15.1	0.00	13.1	0.00	Q
mir-17-3p	66.7	2.89E-05	6.0	8.12E-03	Q
mir-185-3p	5.5	5.65E-03	4.1	1.93E-12	Q
mir-192-5p	3.0	1.52E-04	2.7	8.94E-12	Q
mir-194-1-5p	9.7	2.94E-10	7.3	5.31E-09	Q
mir-194-2-5p	8.7	2.60E-10	4.9	7.26E-08	Q
mir-196b-5p	3.9	6.59E-12	10.9	4.83E-13	Q
mir-19a-3p	13.1	1.56E-08	3.2	4.26E-03	Q
mir-19b-1-3p	19.9	2.87E-08	6.7	5.58E-05	Q
mir-19b-2-3p	19.3	1.98E-08	6.4	3.44E-05	Q
mir-20a-5p	20.2	3.89E-09	11.7	1.32E-07	Q
mir-20b-5p	19.5	1.78E-10	19.8	0.00	Q
mir-223-3p	5.7	0.00	4.4	5.61E-08	Q
mir-26b-5p	2.1	2.54E-04	2.5	0.00	Q
mir-28-5p	−17.0	5.26E-09	−13.9	1.78E-06	NF
mir-29b-1-3p	15.0	2.20E-13	5.2	1.24E-05	Q
mir-29b-2-3p	14.7	6.59E-13	5.8	9.28E-07	Q
mir-324-5p	4.5	1.44E-06	4.7	6.02E-05	Q
mir-340-3p	−13.7	1.48E-06	−30.2	2.19E-04	NF
mir-361-5p	4.0	5.11E-08	3.9	3.48E-08	Q
mir-451a-5p	−6.5	5.56E-68	−4.8	4.41E-12	NF
mir-486-1-3p	−9.2	8.16E-20	−5.8	3.23E-26	NF
mir-486-2-3p	−8.7	3.04E-18	−6.4	2.40E-35	NF
mir-495-3p	−13.5	6.11E-09	−44.3	1.29E-06	NF
mir-502-3p	−6.1	8.01E-03	−2.4	4.58E-05	NF
mir-543-3p	−32.5	1.14E-02	−14.6	2.84E-04	NF
mir-652-3p	−15.0	8.76E-45	−17.8	1.97E-05	NF
mir-885-5p	−20.5	4.21E-02	−9.2	2.29E-03	NF
mir-93-5p	4.5	6.26E-06	4.3	0.00	Q
mir-98-5p	−4.6	8.03E-05	−4.0	1.54E-10	NF

As library preparation method had the greatest effect upon the miRNAs detected, RT-qPCR was carried out on ten miRNAs differentially detected between NF and Q, selected based on read distribution (let-7d-3p, let-7 g-5p, mir-10b-5p, mir-16-5p, mir-16-2-3p, mir-142-3p, mir-26b-5p, mir-223-3p, mir-451a and miR-93-5p). The RT-qPCR validation was carried out on MagnaZol and miRNeasy RNA and Spearman correlation coefficient was calculated between 1/Cq values and number of reads (Table 3). QIAseq libraries showed a significant correlation of 0.73 in MagnaZol RNA and 0.72 in miRNeasy RNA. NEXTflex libraries showed a significant correlation of 0.66 in miRNeasy RNA and a non-significant correlation of 0.59 in MagnaZol RNA. Scatter plots for Table 2, showing 1/Cq vs log(Reads), are provided in Additional file 5. This suggests that quantification based upon reads from QIAseq libraries is closer to the absolute values.

**Table 3:** Spearman Correlation between RT-qPCR and sequencing data

	mNF	mQ	MNF	MQ
Spearman Correlation Coefficient	0.66	0.72	0.59	0.73
p-value/ significance (* *p* < 0.05)	0.04/ *	0.02/ *	0.08/ ns	0.02/ *

### miRNA detection with increasing raw read depth

**Fig. 4:**
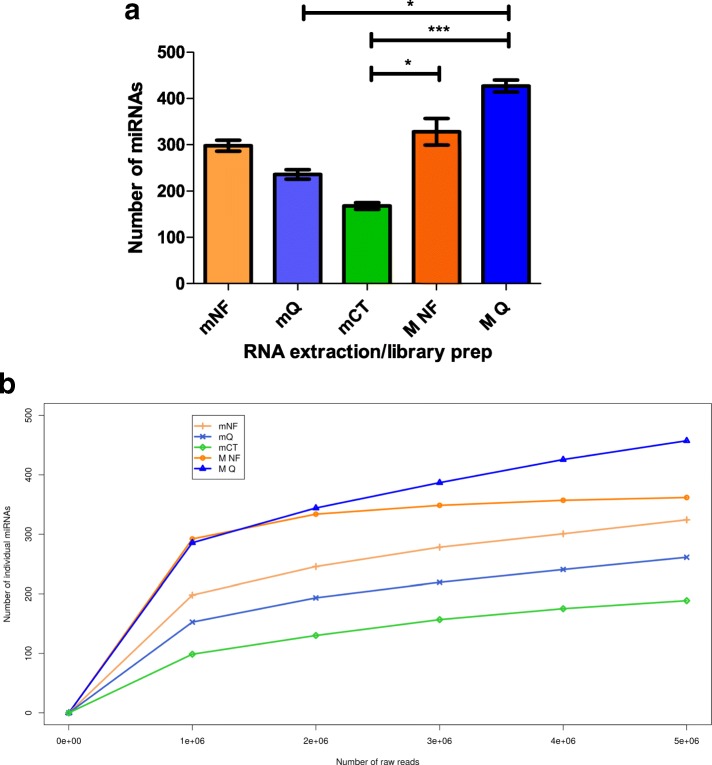
Effect of RNA extraction on miRNA diversity. **a** Number of individual miRNAs detected from an equal number of reads (5 million). miRNAs with a minimum of 2 RPM were counted, error bars show standard error of the mean (n = 6), significance determined by applying Friedman test and Dunn’s multiple comparison test. * p < 0.05 ***p < 0.001 where no p-value shown there was no significance. **b** Number of different miRNAs detected (minimum 10 reads) with increasing read depth up to 5,000,000 reads

In addition to variable library diversity, the differing proportions of reads mapping to miRNAs (Fig. 2) will affect the number of miRNAs detected from a given number of raw reads. Therefore, RNA extraction and library preparation combinations were downsampled to 5 million total reads (Fig. 4a). The most individual miRNAs were detected in libraries prepared from MagnaZol RNA, with MQ detecting an average of 428 miRNAs and MNF detecting an average of 328 miRNAs (all miRNAs detected and the number of reads for each are provided in Additional file 6). From miRNeasy RNA, mNF detected an average of 298 miRNAs, mQ detected an average of 254 miRNAs and mCT detected an average of 168 miRNAs. Friedman and Dunn’s Multiple Comparison testing showed that there were significant differences between MQ and mQ, MNF and mCT and MQ and mCT. To further investigate the effect of sequencing depth upon detection of miRNAs with each protocol, raw reads were downsampled incrementally from 5 million to 1 million reads and the number of miRNAs detected with > 10 reads plotted (Fig. 4b). For all the protocols, 1 million reads represents a reasonable minimum target sequencing depth, with more than half of all of the miRNAs observed at 5 million raw reads already detected. Whilst in most cases, the number of individual miRNAs increased with increased sequencing depth, MNF reached a plateau at ~ 2 million reads, showing that the total number of miRNAs present in this library had been detected. This indicates that when using NEXTflex library kits on plasma, 2 million reads is the maximum sequencing depth required. However, if maximal detection of lowly expressed miRNAs is required, sequencing of an MQ library to a greater read depth is recommended.

### Clustering of groups

**Fig. 5:**
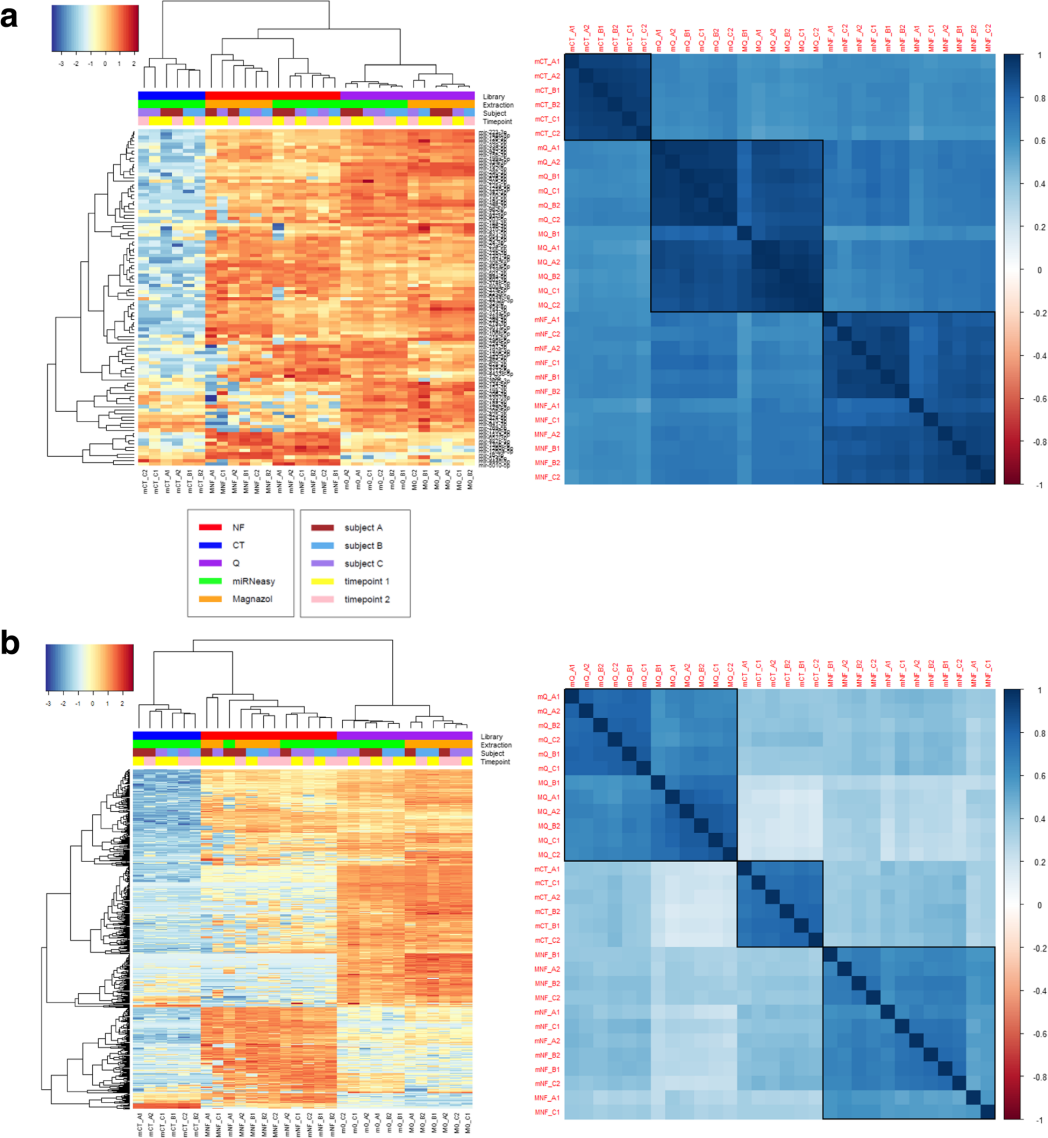
Comparison of RNA extraction and library preparation protocols. **a** Left panel: Hierarchical clustering based on 100 most differentially detected mature miRNAs sequences (≥10 reads from 550,000 reads mapping to miRNAs). Right panel: Correlation matrix of pairwise comparisons of miRNA expression between all RNA extraction/library preparation combinations. **b** Left panel: Hierarchical clustering based on 500 most differentially detected miRNA isomiR sequences (≥2 reads from 550,000 reads mapping to miRNAs). Right panel: Correlation matrix of pairwise comparisons of isomiR expression between all RNA extraction/library preparation combinations. The three main clusters in the Left panels and blocks in the Right panels demonstrate the similarity between libraries of the same type and are subdivided according to RNA extraction method

Hierarchical clustering was carried out on the top 100 most differentially detected mature miRNAs (Fig. 5a, left panel) and the top 500 most differentially detected isomiR sequences (Fig. 5b, left panel). In both cases, samples clustered firstly according to library preparation method and then by RNA extraction method. For both mature miRNAs and isomiRs, mQ most consistently grouped the two samples from the same individual together. The correlation matrices (Fig. 5, right panel) demonstrate that the miRNAs detected by a specific library preparation method are very consistent, although some variability is introduced by different RNA extraction methods. This suggests that any single protocol can be effective for detection of differential miRNA expression, but that comparisons between protocols should be avoided.

### Further considerations

Whilst the varying bias observed with the three library preparation kits described here is likely introduced largely during adapter ligation [8, 9], other kits are available which allow small RNA libraries to be prepared without a ligation step. The CATS Small RNA-seq Kit (Diagenode) and SMARTer smRNA-Seq Kit (Takara Bio) both make use of 3′ polyadenylation and 5′ template switching to enable ligation-free library preparation. Although analysis of libraries prepared from synthetic RNA miRNA pools suggests that ligation-free library preparation has less bias than adapter ligation based methods [23, 24], Dard-Dascot, et al. (2018) have shown that bias still remains when using these methods [21]. Perhaps further optimisation of reaction conditions, which have been shown to have a major impact on bias [11], may enable an additional reduction in bias in the NEXTflex protocol beyond that provided by degenerate adapters. Despite having fixed sequence adapters, the reaction chemistry adopted in the QIAseq protocol enabled preparation of libraries with the least bias in our study, as judged by diversity and correlation with RT-qPCR.

The small size difference between miRNA-containing library products and adapter dimer have meant that to date most protocols require a gel purification step. This is a laborious process which can also introduce more bias [30]. The CT and Q library protocols have minimised background product sufficiently to enable gel-free size selection, while at the RNA concentrations we obtained from plasma, the NF requires a gel purification step. It is possible that this gel purification step contributed to the lower diversity in NEXTflex libraries due to loss of lowly expressed miRNAs.

As with the laboratory protocol it is critical that a consistent data analysis pipeline is applied, with small differences in the permitted number of mismatches and length variations from miRBase having a significant effect upon the miRNAs detected. For example, the online QIAGEN Data Analysis Center reported many lowly expressed miRNAs not represented by any full length mature sequences. The parameters used for assigning reads to a miRNA, particularly extensions at the 3′ end will also influence the range of isomiRs detected. Use of individual isomiRs can assist with clustering of samples and might identify a specific sequence that could act as a more effective biomarker than the total expression of the miRNA it represents.

## Conclusions

Whilst all three library preparation kits investigated in this study can reliably detect miRNAs, we have demonstrated that choice of library kit has the most significant effect on the miRNA profiles detected, however the RNA extraction method must also be considered. Both RNA extraction and library preparation methods introduce greater variation than the biological variation between individuals. Of the three library kits, QIAseq had the highest miRNA diversity from a fixed number of reads mapping to miRNAs and correlated most closely to RT-qPCR. QIAseq libraries prepared on MagnaZol RNA had a significantly higher proportion of reads mapping to miRNAs than those on miRNeasy RNA and exhibited a significantly higher number of individual miRNAs from a fixed number of reads. Therefore, we would recommend using QIAseq library preparation kits on RNA extracted using MagnaZol.

## Methods

### Plasma preparation

Subject recruitment was previously described by McGrath et al (2016) [25]. A fasting blood sample, including a sample anticoagulated with EDTA for the separation of plasma, was collected from all participants at baseline and week 4. All bloods were centrifuged for the isolation of plasma within 2 h of being drawn and stored at − 80 °C.

### RNA extraction and quantification

Total RNA was extracted from 600 μl plasma using MagnaZol™ cfRNA Isolation Reagent (Bioo Scientific) and from 200 μl plasma using miRNeasy Serum/Plasma Kit (QIAGEN) following manufacturers’ instructions. To confirm the presence of miRNAs, samples were quantified using the Qubit™ microRNA Assay Kit (Thermo Fisher).

### Library preparation and sequencing

Libraries were prepared from 5 μl of miRNeasy RNA using NEXTflex® Small RNA Sequencing Kit v3 for Illumina® Platforms (Bioo Scientific) and QIAseq miRNA Library Kit (QIAGEN) and from 2 μl miRNeasy RNA using CleanTag™ Small RNA Library Prep Kit (TriLink), following each of the manufacturers’ instructions. Additionally, libraries were prepared from 5 μl MagnaZol RNA using NEXTflex® Small RNA Sequencing Kit v3 for Illumina® Platforms (Bioo Scientific) and QIAseq miRNA Library Kit (QIAGEN). Library concentrations were measured using Qubit™ dsDNA HS Assay Kit (Thermo Fisher). Quality and concentration of libraries were determined by Fragment Analyzer (Advanced Analytical). Libraries were sequenced on a NextSeq 500 System (Illumina).

### Data analysis

CLCBio Genomics workbench v10.1.1 (QIAGEN) was used to trim FastQ files and align sequences to miRBase release 21 [31–35], allowing 2 mismatches and length within 2 nucleotides of the mature sequence. 5p and 3p mature miRNAs were treated independently in further analyses. The effect of using UMIs was analysed using the QIAGEN Online Data Analysis Center with default settings. Non-coding RNA databases were downloaded from Ensembl using Biomart (https://www.ensembl.org/biomart/martview/) or from the SILVA rRNA database (https://www.arb-silva.de/). Various data analyses were performed in R, including plotting of heatmaps with heatmap3 version 1.1.1 (https://CRAN.R-project.org/package=heatmap3) [36]. Correlation and matrix plotting was performed with the package “corrplot” Version 0.84 [37], available from https://github.com/taiyun/corrplot. For these analyses genes were filtered for those with > 10 cpm and presence in > 20 samples (for isomiR analysis cpm > 2, presence in > 10 samples).

To detect potential plant miRNAs, reads that did not map to human microRNAs were aligned using BLAST with plant mature miRNA sequences downloaded from miRBase Release 22. Sequences with 100% identity, > 17 nucleotides in length were retained.

Analysis of miRNAs differentially detected between RNA extractions, library preparations and timepoints was performed on an equal number of reads mapping to miRNAs in CLC Genomics Workbench using two group paired comparisons with proportion-based statistical analysis performed by applying Baggerley’s test to all pairs and calculating Bonferroni *p*-values. miRNAs > 2 fold differentially detected and with Bonferroni corrected p-values < 0.05 were compared in Venny 2.1.0 [38] to detect miRNAs consistently altered in both extractions or library preparations.

### RT-qPCR

Reverse transcription (RT) and qPCR reactions were prepared on MagnaZol and miRNeasy RNA using TaqMan Advanced miRNA Assays (Thermo Fisher) following the manufacturer’s protocols, with the PCR reaction volume minimized from 15 μl to 2 μl using an Echo 525 Liquid Handler (Labcyte). RT-qPCR was performed on a LightCycler® 480 Instrument II (Roche).

## Additional files

Additional file 1: Workflow highlighting key differences between RNA extraction and library preparation kits. (PPTX 43 kb)

Additional file 2: Sequences matching plant miRNAs. (CSV 505 kb)

Additional file 3: Abundance of potential plant miRNAs. **a.** Number of reads mapping to each potential plant miRNA ((reads/raw reads)x5E6) in individuals A, B and C before and after change in diet to increased plant content. miRNAs occurring in > 1 protocol in an individual are coloured. **b.** Total number of potential plant miRNA reads in each individual before and after change in diet. (PPTX 304 kb)

Additional file 4: All differentially detected miRNAs. **a.** Differentially detected miRNAs between library preparations (NEXTflex and QIAseq) in MagnaZol and miRNeasy extractions. Positive fold change indicates a higher detection in QIAseq and negative fold-change indicates higher detection in NEXTflex. **b.** Differentially detected miRNAs between RNA extractions (MagnaZol and miRNeasy) in NEXTflex and QIAseq library preparations. Positive fold change indicates higher detection in MagnaZol, negative fold change indicates higher detection in miRNeasy. (XLSX 29 kb)

Additional file 5: Scatterplots for correlation between sequencing data and RT-qPCR data. Log(reads) for both NEXTflex and QIAseq were plotted against 100x(1/Cq) for (a) miRNeasy and (b) MagnaZol RNA. (PPTX 82 kb)

Additional file 6: Lists of miRNAs detected in all libraries with 5 million raw reads, including number of reads for each miRNA. (XLSX 172 kb)

## Data Availability

The datasets generated and analysed during the current study are available in the GEO repository, accession number GSE118125.

## Abbreviations

CT: CleanTag
M: MagnaZol™ cfRNA isolation reagent
m: miRNeasy Serum/Plasma extraction reagent
miRNA: MicroRNA
NF: NEXTflex
NGS: Next generation sequencing
Q: QIAseq
rRNA: Ribosomal RNA
RT: Reverse transcription
small RNA-Seq: Small RNA Sequencing
UMI: Unique molecular index
