# Supplementary material for: A comparison of RNA extraction and sequencing protocols for detection of small RNAs in plasma
Source: BMC Genomics. 2019 Jun 3;20:446. doi: 10.1186/s12864-019-5826-7 (PMC6547578; doi:10.1186/s12864-019-5826-7)
Supplement: Supplementary file 1 — Workflow highlighting key differences between RNA extraction and library preparation kits. (PPTX 43 kb) [file 12864_2019_5826_MOESM1_ESM.pptx]

## Slide 1
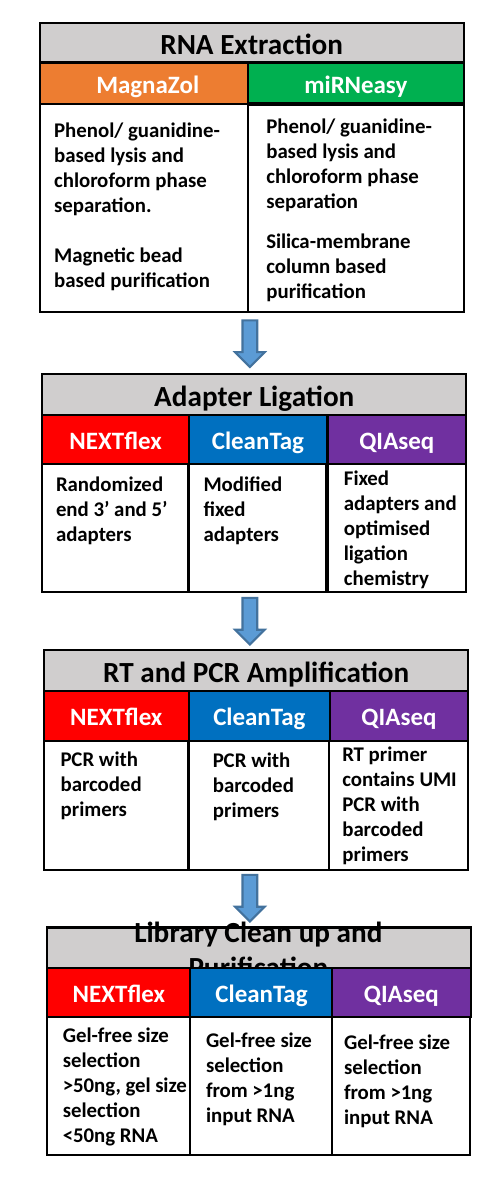

RNA Extraction
MagnaZol
miRNeasy
Phenol/ guanidine-based lysis and chloroform phase separation
Silica-membrane column based purification
Phenol/ guanidine-based lysis and chloroform phase separation.
Magnetic bead based purification
Adapter Ligation
NEXTflex
CleanTag
QIAseq
Fixed adapters and optimised ligation chemistry
Randomized end 3’ and 5’ adapters
Modified fixed adapters
RT and PCR Amplification
NEXTflex
CleanTag
QIAseq
RT primer contains UMI
PCR with barcoded primers
PCR with barcoded primers
PCR with barcoded primers
Library Clean up and Purification
NEXTflex
CleanTag
QIAseq
Gel-free size selection >50ng, gel size selection <50ng RNA
Gel-free size selection from >1ng input RNA
Gel-free size selection from >1ng input RNA
