# Supplementary figures and images for: A comparison of RNA extraction and sequencing protocols for detection of small RNAs in plasma
Source: BMC Genomics. 2019 Jun 3;20:446. doi: 10.1186/s12864-019-5826-7 (PMC6547578; doi:10.1186/s12864-019-5826-7)

## Slide 1
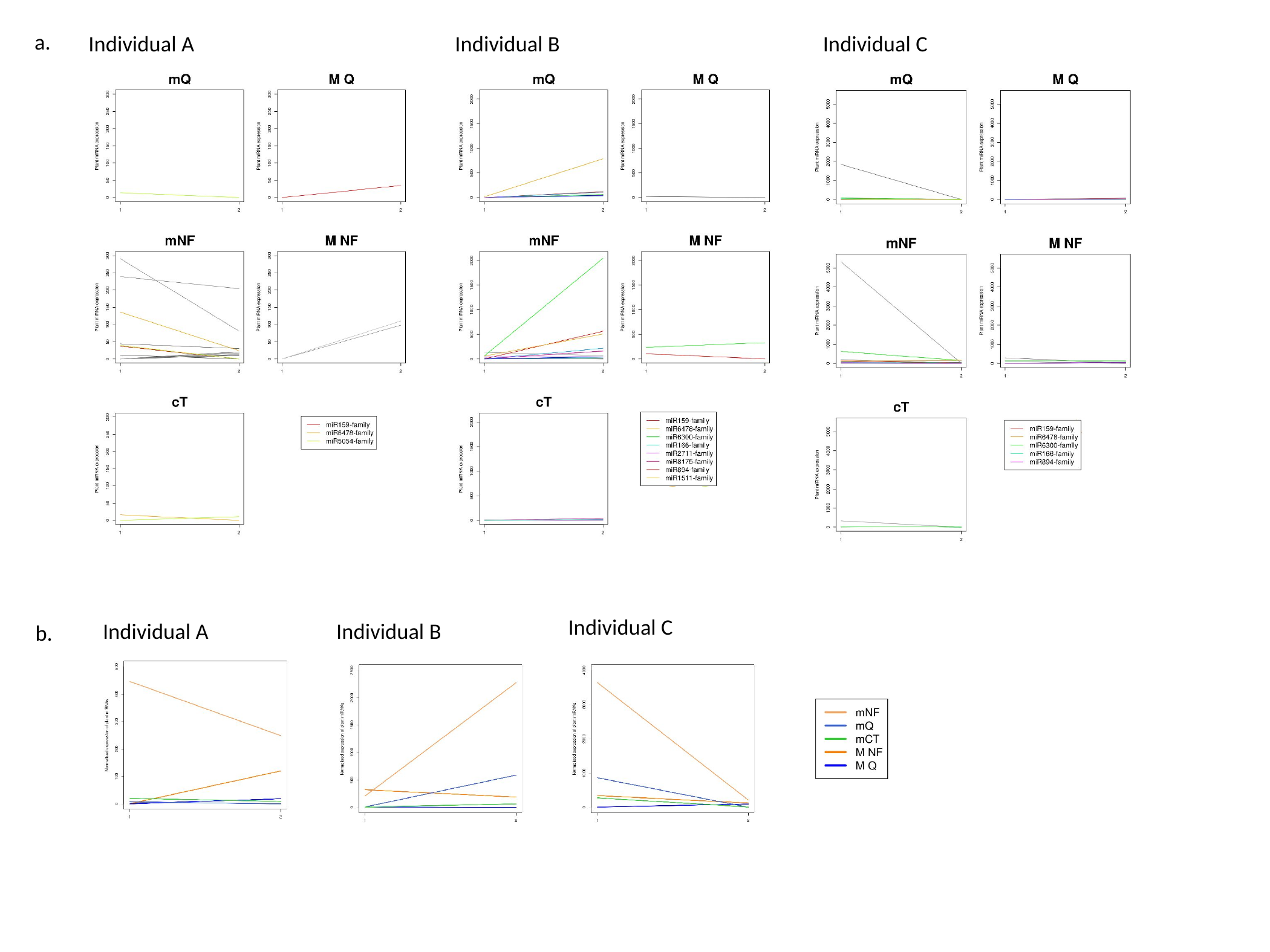

a.
Individual A
Individual B
Individual C
Individual C
Individual A
Individual B
b.

Supplement: Supplementary file 3 — Abundance of potential plant miRNAs. a. Number of reads mapping to each potential plant miRNA ((reads/raw reads)x5E6) in individuals A, B and C before and after change in diet to increased plant content. miRNAs occurring in > 1 protocol in an individual are coloured. b. Total number of potential plant miRNA reads in each individual before and after change in diet. (PPTX 304 kb) [file 12864_2019_5826_MOESM3_ESM.pptx]

## Slide 1
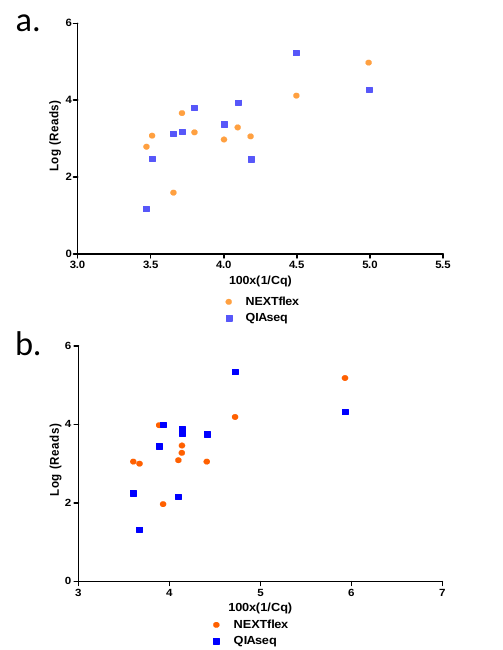

a.
b.

Supplement: Supplementary file 5 — Scatterplots for correlation between sequencing data and RT-qPCR data. Log(reads) for both NEXTflex and QIAseq were plotted against 100x(1/Cq) for (a) miRNeasy and (b) MagnaZol RNA. (PPTX 82 kb) [file 12864_2019_5826_MOESM5_ESM.pptx]
